# Indexing extravascular lung water to predicted body weight increases the correlation with lung injury score in patients with acute lung injury/acute respiratory distress syndrome: a prospective, multicenter study conducted in a Japanese population

**DOI:** 10.1186/cc9562

**Published:** 2011-03-11

**Authors:** H Fukushima, T Seki, Y Urizono, M Hata, K Nishio, K Okuchi

**Affiliations:** 1 Nara Medical University Hopital, Kashihara City, Japan

## Introduction

Since predicted body weight derived from height and gender reflects lung size better than actual body weight, it is reported that extravascular lung water indexed to the predicted body weight (EVLWIp) is more closely correlated with severity of illness and mortality than EVLW indexed to the actual body weight (EVLWIa). However, the usefulness of EVLWIp has not been evaluated in a multicenter study or in the Asian population.

## Methods

We conducted a prospective, multicenter observational study in Japan with the following inclusion criteria: adult (≥18 years) patients needing mechanical ventilation, PaO_2_/FiO_2 _ratio below 300, and acute bilateral infiltrates in both lung fields on chest X-ray. The diagnosis of acute lung injury/acute respiratory distress syndrome (ALI/ARDS) was based on peer review. Predicted body weight was calculated as 50 (for male) or 45.5 (for female) +0.91(centimeters of height -152.4). The normal range of body mass index (BMI) was defined as 18.5 to 22.9. Obesity was defined as BMI ≥25. Data are presented as medians and interquartile ranges (IQR). A Wilcoxon's rank sum test and the Mann-Whitney test were used to compare the values, and correlations were analyzed using Spearman's rank correlation coefficient. Statistical significance was tested at a level of 0.05.

## Results

Seventy-eight patients with ALI/ARDS were enrolled. The values of EVLWIp (17.1 ml/kg; IQR, 12.9 to 21.4) were not different from EVLWIa (16.6 ml/kg; IQR, 12.3 to 21.7). Although the overall correlation with APACHE II score, SOFA score, or mortality was not stronger for EVLWIp compared with EVLWIa, in patients weighing under or over the normal range (BMI <18.5 or BMI ≥23, 41 cases), EVLWIp was more closely correlated with Lung Injury Score (LIS) than ELWIa (EVLWIa; *r*_s _= 0.228, *P *= 0.152 vs. EVLWIp; *r*_s _= 0.333, *P *= 0.033). Furthermore, in patients with BMI ≥25 (19 cases), the correlation of EVLWIp with LIS was much higher (*r*_s _= 0.611. *P *= 0.005) compared with EVLWIa (*r*_s _= 0.283, *P *= 0.240).

## Conclusions

Even in the Japanese population, EVLWIp is more highly correlated to the LIS, especially in obese patients.
